# Supplementary material for: Pregnant women and male partner perspectives of secondary distribution of HIV self-testing kits in Uganda: A qualitative study
Source: PLoS One. 2023 Feb 14;18(2):e0279781. doi: 10.1371/journal.pone.0279781 (PMC9928124; doi:10.1371/journal.pone.0279781)
Supplement: S1 Appendix — (DOCX) [file pone.0279781.s002.docx]

**SUPPLEMENTAL APPENDIX A: ADDITIONAL SUPPORTING QUOTES**

**Table I.** Quotes about the advantages of HIVST and acceptability of secondary distribution

| **Advantages of HIVST** | ***Quotes*** |
| --- | --- |
| **Privacy of HIVST** | *“I would like to use it because it keeps your results secretive in that if you self-test when you are alone, you get to know your status than testing in the public facility because if they disclose to you your results… everyone else can get to know about your status whether you are HIV positive or not.” HIV-negative man, age 30, FGD*  *“It is easy to use and share the results with only your family members, because with the facility testing, all community members can get to know the status of someone.” HIV-negative man, age 34 years, IDI* |
| **Benefits the health of entire family** | *“It helps to keep our families safe and also get to know our HIV status.” HIV-negative man, age 37, FGD* |
| **Ability to test new extra-marital partners** | *“I would like to use it because I may get a side dish and I would like to know her HIV status before we go further.” HIV-negative man, age 37, FGD* |
| **Acceptability of Secondary Distribution of HIVST** | ***Quotes*** |
| **Some men were supportive of receiving HIVST kits from their partners** | *“That kit is not new to me because my wife brought it to me when she was pregnant and I used it, it is really a good kit and people should not be worried about it…if it shows two signs, you are free to go to the health worker for confirmatory testing.” HIV-negative man, age 27, FGD*  *“Obviously the woman will feel good because she would want to know her male partners’ status and this gives her courage and strength if she brings the kit and she will explain it to you on how it is used and to men who don’t want to go to the clinic…It would be better for a woman to bring it to me at home because we men always allege that we are busy with work.” HIV-negative man, age 25, IDI* |
| **Some women expressed confidence and interest in delivering the kit to their partners** | *“I don’t see any challenges or concerns towards delivering the kit to my partner… it is all about giving a genuine explanation to your man like there is new system which introduced for testing HIV and if you are in good terms with your man, he will ask you, where is it? Where did you get it? … I would definitely tell him that when I went to the clinic for ANC, we were sensitized about it.” Woman, unknown HIV status, age 34, IDI* |
| **Secondary distribution can only work if the couple has “mutual understanding”** | *“It depends on the mutual understanding between you and your partner because not every person with a wife at home are in good terms, you can be with a partner at home but when you are not friends so she finds it very hard to persuade you to use the kit but if you are in good terms, it is more easier for her because she will just explain it to you and you get to understand it. There are some hard men, you know in everything, we the men are the hardest creatures, we take long to accept something because you can be with a woman for long but when she tells you something, you shut her up and ask her not to tell you nonsense.” HIV-negative man, age 25, FGD* |
| **Secondary distribution should be accompanied by phone support from a male HCW** | *“It is good for a woman to take it to her partner but in accompany with a telephone contact of the health worker whom he can contact for more clarification…because the woman may not have understood all the procedures.” HIV-negative man, age 30, FGD* |

**Table II**. Quotes on the barriers of HIVST and secondary distribution

| **Barriers of HIVST** | ***Quotes*** |
| --- | --- |
| **Low awareness of HIVST** | *“I wouldn’t want to use it [HIVST kit] because I don’t understand it very well, I only know the option of removing blood from the finger prick.” -Man, undisclosed status, age 36, FGD*  *“I didn’t know HIV could be detected using saliva.” -HIV-positive woman, age 27, IDI* |
| **Fear of HIV testing without counseling in the event of a positive result** | *“Self-testing is very dangerous and you can die because imagine sitting in your room and self-test, then you come to know your status, you can faint and die but in the hospital, the health worker cannot break the news directly so this cannot worry you and even the counseling you receive.” HIV-negative man, age 22, FGD* |
| **Fear of learning one’s status and facing internalized stigma** | *“Deep inside you, you might be knowing that am HIV positive so even if I go to the facility for HIV testing or not, still am going to die so you rather remain not tested and live by God’s grace.” HIV-negative man, age 31, FGD*  *“Majority prefer to live when they don’t know their HIV status… because in most cases, when you come to know that you are HIV positive, you only think about death.” HIV-negative man, age 37, FGD* |
| **Barriers of HIVST secondary distribution** | ***Quotes*** |
| **Fear of relationship dissolution** | *“Some men do think that if a woman gets to know my status, she will abandon me, so they have that fear. They create a tough situation, where you cannot even approach him and talk about HIV testing.” HIV-positive woman, age 27, IDI* |
| **Fear of blame** | *“I have fear because I would think that he is going to say that it seems I slept with another man who is HIV positive and it’s me who has brought the disease.” HIV-negative woman, age 30, FGD*  *“The bad thing about the kit is that the woman may test HIV negative and you self test HIV positive, she will begin to blame you for having multiple partners and the relationship will end.” HIV-negative man, age 25, FGD* |
| **Lack of confidence in the effectiveness of ART in preventing HIV transmission to partners** | *“In my own thinking, I think there is no positive person who can have sex with a negative person and doesn’t infect that person.” HIV-negative man, age 38, FGD* |
| **Fear of sero-discordance and detrimental consequences on relationship** | *“If am HIV negative and my wife is positive, I cannot abandon her because we have been together for a while but I can ask the health worker what preventive measures I can use so that we remain staying together but not at risk.” HIV-negative man, age 30, FGD* |
| **Concerns about intimate partner violence and accusations of mistrust** | *“When they are giving it to you, you get scared and ask yourself a lot of questions and I would think that he may slap me and ask why I am giving him the self-test kit, it seems she doesn’t trust me.”* HIV-negative woman, age 35, FGD  *“I would like to use it but when it is not given to their female partners to deliver it to them because if a couple uses it from home without receiving any counselling, this can result into domestic violence like fighting so for that reason and since some of us do fear the injection, HIV self-test kit would be a good option but when it is used from the hospital or when the couple is at the hospital together with a health care worker.” HIV-negative man, age 34, FGD*  *“Some men are not simple because you will give it to a woman to take it and get slapped by a man so I would think that we emphasize men to come to the clinics but maybe when she trusts or confides in her male partner but it is not good to give a kit to a woman to take it to her partner, some men are very hard.” HIV-negative man, age 32, FGD* |
| **Concerns about female partners ability explain and interpret HIVST kits** | *“It is good for a woman to take it to her partner but in accompany with a telephone contact of the health worker whom he can contact for more clarification…because the woman may not have understood all the procedures.” HIV-negative man, age 30, FGD* |
| **Concerns about female partners finding out men’s HIV status before them** | *“To some men, it is going to be very hard for them to accept it, he will say that she wants him to self-test and take back the kit to the hospital then the health workers disclose his status to her, he will not use.” HIV-negative man, age 32, FGD*  *“The problem I see with that kit is that, you give it to the woman after being tested but she may be positive but brings me the kit to use but remember she will not tell me her status, but instead put it there for me to self-test and after testing, I may be positive or negative and she will not tell me that she was tested before but she will be interested in knowing my status and if you self-test negative and yet she was tested positive, she can’t tell you the truth because you won’t be knowing how to use it.” HIV-negative man, age 42, FGD* |

**Table III.** Suggestions for linking men to confirmatory testing and counseling

| **Suggestion** | ***Quotes*** |
| --- | --- |
| **Community sensitization** | *“It would be your role as health workers to put in place outreaches in the communities and sensitize the youths or men because we are the difficult population to test for HIV. Let it be a community sensitization, teach us the benefits and draw backs of testing for HIV and let us feel attracted to go for HIV testing.” HIV-negative man, age 22, FGD*  *“It would be so good we have been sensitized about it so if she happens to bring it tomorrow, you already have prior knowledge about it.” HIV-negative man, age 34, FGD* |
| **Counseling** | *“The good thing about the health workers… when you are tested HIV positive, you don’t remain the same so they comfort you that you can also continue with your daily life while positive, giving you [treatment and] whatever you need, you can access it on time from the hospital and even the health workers being friendly to you, it helps you to stay strong.” HIV-positive man, age 27, FGD*  *“It is up to the health workers to follow up the HIVST kit… you can contact the man on the phone and if he declines to pick it, then you can call his wife and … if the man refuses to come for treatment, the health worker is entitled to go to their home and counsels him and it is the responsibility of the hospital to follow up these kits which were delivered to men and if it doesn't do so, something can go wrong.” HIV-negative man, age 25, FGD* |
| **Separate “male-friendly” waiting room** | *“Having a separate waiting room is so important because I cannot seat in the women’s clinic since there are so many people who know me but with a separate room, you will not come to know what is going to happen.” HIV-negative man, age 32, FGD* |
| **Male guide at the clinic** | *“P7: If you give him a leaflet with the contacts of the health worker, he is going to meet, and this [man] should be trained in counseling, talks calmly, this will help him to come to the clinic for care…he will even call that number to confirm and the way the health worker responds to him also determines whether he will link to care.” HIV-negative man, age 30, FGD*  *“It helps so much because if you go somewhere and there is someone to guide you through the process of HIV testing than being stranded because that place will be new to you but if you get someone to help you, it also makes sense.” HIV-negative man, age 30, FGD* |
| **Male peer support counselor who is HIV-positive** | *“What can help such men to link to support and care after self-testing HIV positive is to have a glance at fellow men who are in the same status but in good health condition, like one is HIV positive but he goes to the clinic and access drugs, looks after his family and he is still strong, working and has no signs and symptoms so this will give him courage to also go to the clinic and seek support and care.” HIV-negative man, age 30, FGD* |
| **Extended clinic hours** | *“It will be helpful to men who don’t work on weekends and he will get time to go to the clinic for counseling and other health related services…people should be made aware about it and the time clinics open.” HIV-negative man, age 30, FGD* |
| **Financial incentives, fatherhood skills, and employment training classes** | *“The small financial incentive, I think it can attract men because in most cases, you can invite someone in such a discussion but the first excuse he gives you is being busy at work but if you inform him that he will get transport money, he will come and gain two things like getting knowledge and money and his pockets will be happy.” HIV-negative man, age 30, FGD*  *“The idea of giving out some money for transport will help so many people because some people complain of not having transport but if he comes and gets that money, it will be helpful than using his money meant for food.” HIV-negative man, age 58, FGD* |
| **Fatherhood skills and employment training classes** | *“Many men with women but unemployed and they are not able to look after their spouses so if he impregnates a woman and attends the clinic and receive free fatherhood skills, it will attract so many men coming with their wives to the clinic.” HIV-negative man, age 28, IDI*  *“Educational workshops [will work] because now days jobs are competitive and even those workshops take away stress and what brings worries is poverty but if you have something to do and earn some money, it is the best than having nothing. Even the issue of transport reimbursement to cover up transport costs to the clinic, it will also attract them because he would know that he will use part of the transport money to buy other necessities.” HIV-positive woman, age 24, IDI* |

**Table IV.** Perceptions of alternate HIVST distribution strategies

| **Perceptions on alternate HIVST distribution strategies** | ***Quotes*** |
| --- | --- |
| **Clinic distribution from Healthcare workers** | *“Since I have come to know about it, I feel comfortable when I get it from the health facility because it will not be fake because people have forged so many things.” HIV-negative man, age 28, FGD*  *“That HIVST kit, it needs to use it while in the hospital then afterwards, they counsel you … but if you self-test at home and you come to know that you are positive, you feel heart broken and you can’t even come to the hospital to get counseling.” HIV-negative woman, age 27, FGD* |
| **Pharmacy distribution** | *“You see these kits for pregnancy test… even the self-testing kits will add on the number of people accessing them if you have inform people about it and they will be picking them from the pharmacies.” HIV-negative man, age 28, IDI*  *“The health care workers working in those pharmacies have got ‘lugambo’ [rumor monger].” HIV-negative man, age 25, IDI*  *“The issue is money, those kits are going to be sold by the pharmacy attendants and I will not go to buy a kit when my family doesn’t have what to eat, no way.” HIV-negative man, age 31, FGD* |
| **Community health worker (CHW) distribution** | *“We should get the kits from the clinics where we access HIV treatment but if you give them to the community health workers, it will not work because if you happen to disclose to them your results, they will make a story in the village.” HIV-positive man, age 51, IDI*  *“These community health workers will not give them out to people, they will instead sell them out…. It is not good for [CHWs] to get to know your status, they will spread the information, but the clinic healthcare providers keep it confidential.” Woman unknown status, age 34, IDI* |
| **Phone call from a healthcare worker** | *“ [A phone counselor] will be helpful and the man will come because you call that help line number when you are alone and I think it will help a lot compared to giving the kits to women to take them to men.” HIV-negative man, age 35, FGD* |
| **Home visit from a healthcare worker** | *“If a woman brings me the HIV self-test kit, I will not use it unless she comes with a health worker who has given it to her because if she compels me to use it, we may fight and if she brings it to me, it shows that she doesn't trust me.” HIV-negative man, age 25, FGD*  *“It would be great for the clinician to visit me in the home because he can advise me on certain things like hygiene.” HIV-negative man, age 25, FGD*  *“I would prefer to find the clinician in the hospital for safety reasons because I wouldn't want people to be inquisitive about his visit. HIV-negative man, age 34, FGD*  *“If you are a man, you feel pleased just like me, I would be happy about it and this will show that the healthcare worker minds so much about my life… this even motivates me to come and receive treatment from the clinic.” HIV-negative man, age 35, FGD* |

**SUPPLEMENTAL APPENDIX B: ADDITIONAL DETAILS REGARDING METHODS**

**Methods to project confidentiality**

Personal identifying information for contact and scheduling purposes were retained at the study site by trained qualitative researcher (JM). Participants were given unique identification numbers and all transcripts were stripped of any personal identifiable information. All transcript files were managed and stored on secure servers at the study site and the University of Washington in Seattle. All recording devices were wiped clean after recordings were transcribed. Participants were assured their study information would not be released without their written permission, except if required by Ethical Committees.

**Experience of Qualitative Interviewer**

Author JM holds a Bachelor of Arts Degree in Education from Makerere University in Uganda and certification in Luganda-English translations. He works full-time as a qualitative researcher and is involved in conducting interviews, analyzing and writing analysis reports, contributing to protocol development, and writing abstracts. He is fluent in both written and spoken English and Luganda, the language in which FGDs and IDIs were conducted for this study. JM disclosed his research interests to all study participants as part of the informed consenting process.

**Reflexivity and Rigor of the Qualitative Analysis**

This analysis adhered to standards of rigor through extensive efforts to maximize data quality and ensure interpretations from the data were tightly reasoned and grounded in evidence. A semi-structured interview guide made questions clear without probing, and interviewer (JM) remained open and asked clarifying questions to capture detail and ensure alignment between participant experiences and data captured.

**Interviews**

Interview guides were not formally pilot tested but underwent substantial iteration between researchers at the University of Washington, Harvard University and the Infectious Diseases Institute in Uganda. Interviews were conducted in discreet and convenient locations in the community, where participants felt comfortable speaking about sensitive topics. Data were not collected regarding refusal to participate or dropouts. Transcripts were not returned to participants, and repeat interviews were not carried out.
