# Supplementary material for: Pregnant women and male partner perspectives of secondary distribution of HIV self-testing kits in Uganda: A qualitative study
Source: PLoS One. 2023 Feb 14;18(2):e0279781. doi: 10.1371/journal.pone.0279781 (PMC9928124; doi:10.1371/journal.pone.0279781)
Supplement: Attachment — Submitted filename: Reviewers comment_PLOSONE.pdf [file pone.0279781.s003.pdf]

Pregnant women and male partner perspectives of secondary distribution of HIV self-testing kits in Uganda: A qualitative study.

Congratulations on your study and development of this manuscript for publication. A study of this nature potentially can add to the body of knowledge on strategies to increase uptake of HIV testing among men in SSA. Below I offer comments that could make the manuscript more improved and valuable.

GENERAL COMMENT:
Overall the study was well designed and implemented and the manuscript is well written. However I have few comments below to improve the manuscript.

1. Abstract: I would like to suggest that the authors should be consistent in using the words:" Facilitators vs. motivators". These words are used interchangeable through out the manuscript. See line 62 on page 3, line 112 on pages 5, and line 122 on pages 6, etc.
2. Introduction: The following sentence mentions "…regarding HIVST acceptability, barriers….". Did the authors also assessed "Motivators/facilitators?. If yes, it is not mentioned. See line 96 page 5.
3. Data collection and analysis: Did JM conduct all the interviews alone? What about a note taker? See line 125 on page 6.
4. It seems JM (male interviewer) conducted interviews for women. Could this be a limitation for women to express themselves freely on such sensitive issues to a male interviewer compared to a female interviewer? Could authors clarify on this observation? See line 125 on page 6.
5. In principal translations should be done by a qualified translated. Does this imply that JM is a qualified Translator? Could authors clarify on this observation? See line 129 on page 6.
6. The authors should mention what was the role of using the NVivo software in this study. See line 130 on page 6.
7. The authors mentioned that all FGDs and IDIs were double-coded to assess inter-coder reliability. Could authors provide the results of the inter-coder reliability? See 131 on page 6.
8. The authors also mentioned that they used the Social ecological model to organize the research questions and findings. However, It was very difficult to follow how the authors operationalize their findings and what was presented in Figure II. I suggest that the authors should align their findings and what is presented in the current Figure II. An additional description accompanying the Figure II will be very beneficial to readers. See line 132 on page 6 and line 170 on page 9.
9. The authors reported that they used deductive thematic analysis. The authors should provide justification of using the deductive thematic analysis compared to inductive thematic analysis. See line 134 on page 6.
10. The authors did not mention whether FGDs and IDIs data were analyzed jointly or separately and combined later during interpretation.
11. The authors did not mention how they applied reflexivity and rigor in terms of trustworthness, dependability, credibility and transferability throughout the data

analysis from data collection to interpretation of the findings.

12. Ethical approval: The authors should provide ethics clearance certificate ID for both Ethics committees. See line 137 on page 6.
13. Authors did not mention measures taken to protect confidentiality and anonymity in this study.
14. Results: It was unnecessary to present median & IQR in a qualitative study. Could authors clarify on this observation? See line 144 and 148 on page 7.
15. The following sentence…."did not know HIV could be detected using saliva". I think using the word 'saliva' is not correct in the HIVST perspective. What is used is oral fluid from the mucosa of the mouth. I suggest this should be corrected. See line 176 on page 9.
16. The following quote: *" HIV self-test is like a fishing net…their partners to the hospital to test"*. This quote does not support what is described above. Authors should justify the use of the quote or present a relevant quote. See lines 187-190 on page 10.
17. Conclusion: Is missing in the main manuscript but presented in the abstract. Authors should add their conclusion in the main manuscript.
18. Authors have mentioned among other interventions " financial incentives". Despite existing literature supporting this intervention, I was wondering if this would be sustainable in case of scaling-up to the general population.
19. One of the key barriers for uptake of HIVST is the high cost of buying the HIV self-test kits. I was wondering if the authors assessed this common barrier.
